# Adaptive Differences in Cellular and Behavioral Responses to Circadian Disruption between C57BL/6 and BALB/c Strains

**DOI:** 10.3390/ijms251910404

**Published:** 2024-09-27

**Authors:** Changxiao Ma, Haonan Li, Wenyu Li, Guangrui Yang, Lihong Chen

**Affiliations:** 1 School of Bioengineering, Dalian University of Technology, Dalian 116024, China; changxiaoma@mail.dlut.edu.cn (C.M.); lhn990602@mail.dlut.edu.cn (H.L.); 2 Health Science Center, East China Normal University, Shanghai 200241, China; 51251300109@stu.ecnu.edu.cn; 3 School of Clinical Medicine, Shanghai University of Medicine & Health Sciences, Shanghai 201318, China

**Keywords:** entrainment, circadian disruption, cell, behavior, C57BL/6 mice, BALB/c mice

## Abstract

The regulation of the mammalian circadian clock is largely dependent on heredity. In model animals for circadian rhythm studies, C57BL/6 and BALB/c mice exhibit considerable differences in their adaptation to circadian disruption, yet deeper comparisons remain unexplored. Here, we have established embryonic fibroblast cells derived from C57BL/6 mice (MEF) and BALB/c (BALB/3T3) mice, which have been transfected with the *Bmal1* promoter-driven luciferase (*Bmal1-Luc*) reporter gene. Next, dexamethasone was applied for various cyclic stimulations, which revealed that *Bmal1* bioluminescence of MEF cells was entrained to 24 to 26 h cycles, whereas BALB/3T3 cells have a wider range (22 to 28 h) with lower amplitudes. Behaviorally, BALB/c mice swiftly adapted to a 6-h advance light/dark cycle, unlike C57BL/6 mice. Furthermore, we found the expression of the circadian rhythm gene *Npas2* in BALB/c mice is significantly lower than that in C57BL/6 mice. This observation is consistent with the differentially expressed genes (DEGs) in the intestine and lung tissues of C57BL/6 and BALB/c mice, based on the RNA-seq datasets downloaded from the Gene Expression Omnibus (GEO). In summary, our study uncovers that BALB/c mice possess greater resilience in circadian rhythm than C57BL/6 mice, both cellular and behaviorally, identifying potential genes underlying this difference.

## 1. Introduction

The existence of a mammalian biological clock allows the body to respond to external environmental cues, such as photoperiod, food, temperature, etc. [1,2]. The circadian clock system is intricately governed by the central clock in the suprachiasmatic nucleus (SCN) of the hypothalamus, which is primarily influenced by external light/dark cycles and regulates numerous peripheral biological clocks of body organs via hormonal and neuro-humoral pathways [3]. Glucocorticoids are one of the signals carrying information on light–dark to peripheral clocks [4]. It is rhythmically synthesized and secreted from adrenal glands in an SCN-dependent pattern, and binds to promoters of circadian genes, inducing phase shifts in peripheral clocks including liver, kidney, lung, and cultured fibroblasts [5,6]. The basic timing unit of the circadian clock is the cell, and even individual fibroblasts possess a conserved, cell-autonomous clock. Dexamethasone (DEX), a glucocorticoid hormone analog, is extensively employed to reset and synchronize circadian rhythms by inducing clock gene expression [7].

Mice are the most generally employed mammalian model for investigating circadian rhythm, which is influenced by various characteristics including age, gender, and strain [8]. Evidence revealed that the free-running period (FRP) in constant darkness (DD), which reflects the endogenous circadian clock of nocturnal animals, in BALB/c mice, is typically significantly shorter than that in C57BL/6 mice [9]. In addition, the circadian behavior of BALB/c mice is more flexible in entrainment to various light/dark cycles compared to C57BL/6 mice [10,11]. However, most research on the adaptability differences in circadian clocks from two distinct genetic backgrounds has focused on behavioral rhythms, with scant attention devoted to the cellular level, and the underlying molecular mechanisms remaining elusive.

The molecular mechanism of circadian oscillation is the transcription and translation negative feedback loop composed of core clock genes [12]. Among them, the largest circadian gene (176.68 kb) is *Npas2* (also known as *Mop4*), which encodes the neuronal PAS domain protein 2 [13]. NPAS2 is a paralog to the CLOCK (Circadian Locomotor Output Cycles Kaput) protein, which can functionally replace Clock in binding with BMAL1 (Brain and Muscle Arnt-Like 1, ARNTL1) to regulate the biological clock and is expressed in SCN and peripheral tissues [14]. Extensive studies have shown that NPAS2 refers to fundamental physiological processes that cover circadian rhythm, diabetes, cardiovascular disease, and tumorigenesis [15].

Thus, we utilized repeated periodic stimulation with DEX on MEF and BALB/3T3 cells that inserted the *Bmal1-luc* reporter gene to record the cellular clock. We then analyzed the behavioral rhythms of the two strains of mice. Furthermore, we obtained RNA-seq profiles of various tissues from GEO datasets and verified the differentially expressed *Npas2* between C57BL/6 and BALB/c mice.

## 2. Results

### 2.1. Entrainment Range of MEF and BALB/3T3 to DEX Stimulation T Cycles

**Figure 1:**
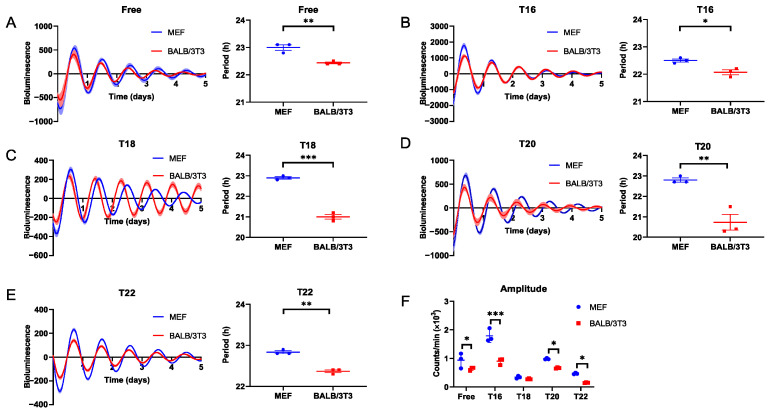
Measurement of the adaptability of MEF and BALB/3T3 cellular circadian rhythms to DEX T cycles < 24 h. (**A**) Free run; (**B**) T16; (**C**) T18; (**D**) T20; (**E**) T22. (**F**) The amplitude of *Bmal1* in MEF and BALB/3T3 cells under different T cycles (n = 3; * *p* < 0.05, ** *p* < 0.01, *** *p* < 0.001).

Our previous studies have demonstrated that BALB/c mice have a wider entrainable range for behavioral rhythm than C57BL/6 mice [10]. Here, to determine adaptive differences between C57BL/6 and BALB/c mice at the cellular level, we explore the entrainment capacity of MEF and BALB/3T3 cells during various stimulation cycles using DEX in vitro. We found that the FRP of BALB/3T3 cells was significantly shorter than that of MEF cells (*p* < 0.05; Figure 1A), and this discrepancy persisted in DEX treatments with T < 24 (Table S1). The bioluminescence periods in MEF and BALB/3T3 cells treated with dexamethasone in cycles of 16 and 18 h (T = 16, 18) were similar to FRP (Figure 1B,C; Table S1). Although the *Bmal1-Luc* period was shortened in dexamethasone-treated BALB/3T3 cells with T = 20 (20.73 ± 0.38 h), it is still clearly not matched to 20 h (Figure 1D, Table S1). However, BALB/3T3 cells could synchronize to the stimulation cycles of T = 22, not MEF cells (Figure 1E, Table S1). During DEX stimuli with T < 24, except for T18, the bioluminescence amplitude of the BALB/3T3 cell was lower than that of the MEF cell (Figure 1F).

**Figure 2:**
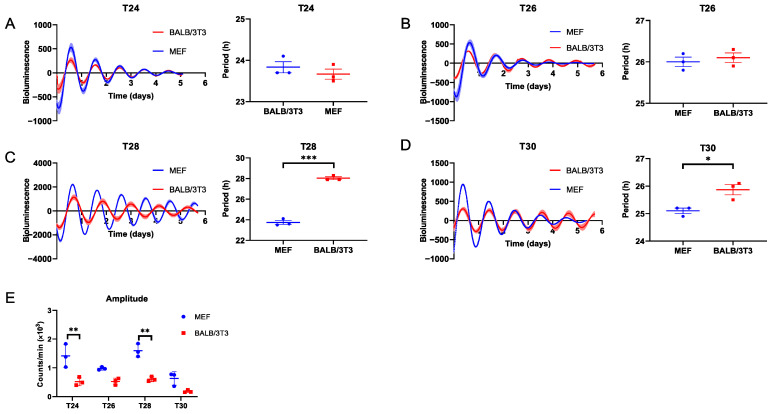
Measurement of the adaptability of MEF and BALB/3T3 cellular circadian rhythms to DEX T cycles ≥ 24 h. (**A**) T24; (**B**) T26; (**C**) T28; (**D**) T30. (**E**) The amplitude of *Bmal1* in MEF and BALB/3T3 cells under different T cycles (n = 3; * *p* < 0.05, ** *p* < 0.01, *** *p* < 0.001).

Moreover, both cell types could be entrained to T24 and T26 (Figure 2A,B; Table S1). Unlike during T26, when exposed to T28, the bioluminescence period of MEF cells could not expand to 28 h as BALB/3T3 cells did but instead followed similar to FRP (23.73 ± 0.19 h; Figure 2C, Table S1). The circadian rhythms of both cell types could not be entrained to T30, even though their bioluminescence periods were significantly longer than FRP (Figure 2D, Table S1). In addition, the circadian amplitude of BALB/3T3 cells was significantly lower than that of MEF cells in the above T cycles (Figure 2E). These data indicate that, in vitro, BALB/3T3 cells exhibit a shorter FRP and lower amplitude compared to MEF cells, as well as a broader entrainment range to periodic DEX stimulation with T cycles (T22–28). Conversely, the adjustability of the biological clock in MEF cells is less flexible, and constrained to a narrower range (T24–26).

### 2.2. Behavioral Rhythms of C57BL/6 and BALB/c Mice in LD and DD Condition

**Figure 3:**
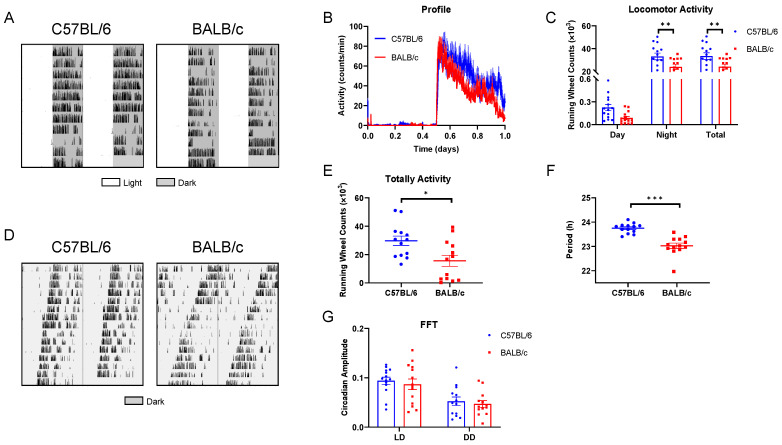
The basal behavioral rhythm in C57BL/6 and BALB/c mice. (**A**) Representative double-plotted actograms of wheel running activity of C57BL/6 and BALB/c mice in LD cycle. (**B**) The average 10-day diurnal activity profile in LD cycles. (**C**) Daytime, nighttime, and total activity levels. (**D**) Double-plotted actograms of wheel running activity of C57BL/6 and BALB/c mice in DD condition. (**E**) Totally activity in DD. (**F**) FRP. (**G**) The FFT in LD and DD (n = 14; * *p* < 0.05, ** *p* < 0.01, *** *p* < 0.001).

The wheel-running activity actograms of C57BL/6 and BALB/c mice in LD were recorded (Figure 3A). Compared with C57BL/6 mice, the nocturnal activity was significantly lower in BALB/c mice, whereas C57BL/6 mice exhibited a higher total activity (Figure 3B,C). In addition, we conducted the behavioral rhythm of mice in DD (Figure 3D), and found the total activity of BALB/c mice was significantly reduced than C57BL/6 mice (Figure 3E), and the FRP of BALB/c mice was significantly shorter than that of C57BL/6 mice (Figure 3F). Furthermore, regardless of LD or DD, the amplitude of FFT (Fast Fourier Transform) revealed no statistical differences in the stability of rhythm between C57BL/6 and BALB/c mice (Figure 3G).

### 2.3. Behavioral Rhythms of C57BL/6 and BALB/c Mice in Phase Shift

**Figure 4:**
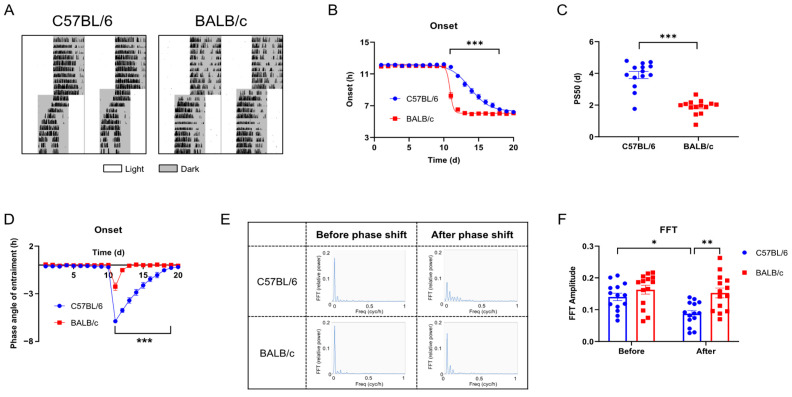
The entrainment of C57BL/6 and BALB/c mice to the +6 shift. (**A**) Representative double-plotted actograms of wheel running activity of mice treated with the + 6 shift. The white bar represents the day, and the gray bar represents the nighttime. (**B**) Quantified activity onsets. (**C**) PS50 values are calculated by activity onset. (**D**) Phase angle of entrainment. (**E**) FFT relative power for 7 days before and after the shift. (**F**) The amplitude of circadian behavior revealed by FFT relative power (n = 14; * *p* < 0.05, ** *p* < 0.01, *** *p* < 0.001).

**Figure 5:**
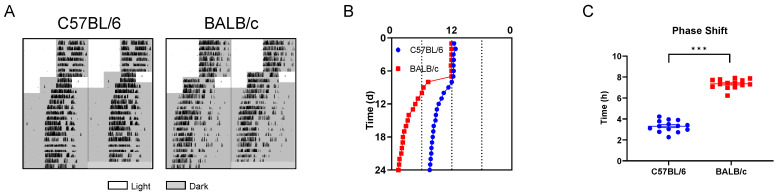
The internal behavioral rhythm of C57BL/6 and BALB/c mice in the +6 shift. (**A**) Representative double-plotted actograms of running-wheel activity of mice in the +6 shift followed by DD. (**B**) Quantification of activity onset. (**C**) The magnitude of the phase shifts after DD (n = 14; *** *p* < 0.001).

To evaluate the entrainment capacity of C57BL/6 mice and BALB/c mice in the light/dark cycle, we advanced LD cycles by 6 h (+6 shift; advance time of lights off by 6 h) (Figure 4A). Compared with C57BL/6 mice, the activity onset of BALB/c mice synchronized to the new LD cycle faster (Figure 4B), and the PS50 (time to reach 50% phase shift) was also lower (*p* < 0.005; Figure 4C). The entrainment phase angle indicated that C57BL/6 mice took approximately 8 days to entrain to the new LD cycle, while BALB/c mice only took about 3 days (Figure 4D). FFT analysis of the 7 days before and after the phase shift revealed that, unlike BALB/c mice, the rhythm stability of C57BL/6 mice was significantly reduced after the +6 shift (Figure 4E,F). To verify whether the rapid adaptation to the +6 shift in BALB/c mice was an illusion caused by masking [16,17], we released the C57BL/6 and BALB/c mice into DD 2 days after administering the shift treatment to analyze the internal circadian rhythm (Figure 5A). We found that the fast entrainment to the new LD cycle in BALB/c mice was driven by the internal biological clock (Figure 5B), which was further confirmed by the significantly larger phase advance in BALB/c mice after the +6 shift, rather than C57BL/6 mice (Figure 5C). The findings demonstrate that, consistent with cellular-level data, BALB/c mice exhibit lower locomotor activity, a shorter free-running period (FRP), and faster adaptation to LD shifts compared to C57BL/6 mice.

### 2.4. Expression of Npas2 in C57BL/6 and BALB/c Mice Was Compared

**Figure 6:**
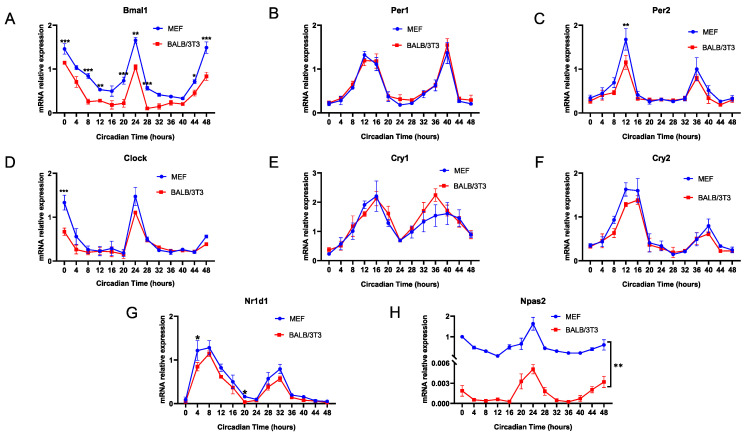
Expression rhythms of core clock genes in MEF and BALB/3T3 cells. (**A**) *Bmal1*; (**B**) *Per1*; (**C**) *Per2*; (**D**) *Clock*; (**E**) *Cry1*; (**F**) *Cry2*; (**G**) *Nr1d1*; (**H**) *Npas2* (n = 3; * *p* < 0.05, ** *p* < 0.01, *** *p* < 0.001).

Next, we explored the differences in the expression rhythms of various classic clock genes in MEF and BALB/3T3 cells, and the results showed that the mRNA rhythmic expression of *Bmal1* in BALB/3T3 cells was always lower than that in MEF cells (Figure 6A). In addition, the expression of other core circadian clock genes, including *Per1/2*, *Clock*, *Cry1/2*, and *Nr1d1*, did not differ between the two cell lines, even though the expression of some circadian genes in BALB/3T3 cells was less than that in MEF cells at individual time points (*Per2* at CT12; *Clock* at CT0; *Nr1d1* at CT4) (Figure 6B–G). Notably, as an alternative to *Clock*, the oscillatory rhythm of *Npas2* is similar to *Bmal1*, peaking around CT0 [14], and expression of *Npas2* in BALB/3T3 cells was significantly lower than that in MEF cells at any time point (Figure 6H). The expression phase of these circadian genes in cells of the two strains did not differ.

**Figure 7:**
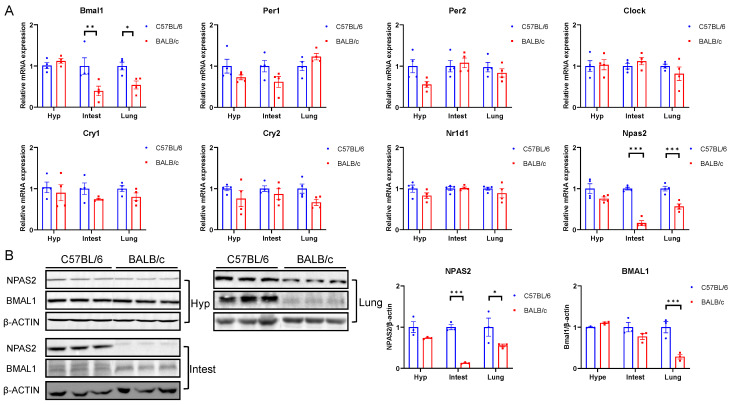
Expression of core clock genes in C57BL/6 and BALB/c mice tissue. (**A**) mRNA (n = 4) and (**B**) western blotting (n = 3) and corresponding data statistics for expression of core clock genes in the hypothalamus, intestine, and lung (* *p* < 0.05, ** *p* < 0.01, *** *p* < 0.001).

Since we had already analyzed the expression rhythm of *Npas2* in both MEF and BALB/3T3 cells, we focused on a representative time point for tissue sampling. Our primary goal was to examine the differences in *Npas2* expression levels between the two mouse strains across various tissues. Next, we analyzed the expression of clock genes in major central and peripheral tissues between C57BL/6 and BALB/c mice and found that the mRNA expression of *Bmal1* in the lung and intestine of BALB/c mice was significantly lower than that of C57BL/6 mice (Figure 7A), but this disparity was reversed in the heart (Figure S1A). In C57BL/6 and BALB/c mice, consistent with the performance in cells, there was no difference in the expression of other classical circadian clock genes in various tissues (Figure 7A), though the expression of *Per1* within the brown adipose tissue (BAT) and Cry2 within the heart in BALB/c mice were significantly lower than those of C57BL/6 mice (Figure S1B–G). Furthermore, the expression of *Npas2* in the hypothalamus, intestine, lung, liver, BAT, and heart of BALB/c mice was significantly lower than that of C57BL/6 mice (Figure 7A and Figure S1H). In addition, except for the hypothalamus, the protein expression of NPAS2 in the liver, intestine, and lung of BALB/c mice were still significantly lower than that of C57BL/6 mice (Figure 7B). The difference in the expression of classic clock genes in cells and tissues between C57BL/6 and BALB/c mice confirms that the expression of *Npas2* in BALB/c mice is significantly lower than that in C57BL/6 mice. Although the expression of *Bmal1* in BALB/c mice is also observed to decline, the significant degree is much lower than that of *Npas2*. In addition, previous studies have demonstrated that the absence of *Npas2* and *Bmal1* genes in C57BL/6 mice can rapidly match LD shifts [18,19]. This suggests that *Npas2* is a potential key gene that regulates the differences in circadian rhythm adaptation between C57BL/6 and BALB/c mice.

### 2.5. DEGs in Various Tissues of C57BL/6 and BALB/c Mice

To further probe the DEGs related to circadian rhythm between C57BL/6 and BALB/c mice, we obtained RNA-seq data of the whole brain, intestine, and lung of wild-type (WT) C57BL/6 and BALB/c mice from the GEO database and analyzed the DEGs therein. First, we found 79 DEGs in the brain, while there are no genes that directly regulate circadian rhythms (Figure S2A,B). Then, we obtained 133 DEGs in the intestine of C57BL/6 and BALB/c mice, including 51 upregulated DEGs and 82 downregulated DEGs, and involved circadian rhythm regulation gene *Dbp*, *Npas2*, and *Bmal1* (Figure S2C). Among the DEGs in C57BL/6 and BALB/c mice, gene ontology (GO) enrichment analysis has revealed pathways associated with retinol metabolism, biological oxidations, circadian rhythm, and those related to inflammatory response. Notably, circadian rhythm pathways feature key genes such as *Bmal1* and *Npas2* (Figure S2D). For the lung tissue, compared to C57BL/6 mice, a total of 137 DEGs were upregulated, and 249 DEGs were downregulated in BALB/c mice and results are presented in the volcano plot (Figure S2E).

The significant GO pathways include immune response-related pathways (including activation of innate immune response and production of molecular mediator involved in inflammatory response), biological regulation of metabolism (such as neutrophil degranulation and neutrophil degranulation), gene expression and regulation (including MHC class II protein complex assembly and epigenetic regulation of gene expression), and circadian regulation of gene expression (including *Bmal1* and *Npas2*) (Figure S2F). These data suggest that there are differences between C57BL/6 and BALB/c mice in immune response, gene expression, metabolism, cellular function, and signal transduction. Notably, different co-DEGs are observed in various tissues, with *Npas2* and *Bmal1* being the most likely candidates, exhibiting lower expression levels in BALB/c mice compared to C57BL/6 mice.

## 3. Discussion

In this study, we have explored the adaptability of C57BL/6 and BALB/c mice to external periodic stimuli and mechanisms from both cellular and behavioral perspectives. There are significant differences in the biological clock of mice with various genetic backgrounds. Our previous research found that BALB/c mice have a wider entrainment range for non-24-h T cycles than C57BL/6 mice [10]. To further delve into the more intrinsic mechanisms underlying the differences in behavioral rhythms between C57BL/6 and BALB/c mice, we established embryonic fibroblast cell lines of C57BL/6 (MEF) and BALB/c mice (BALB/3T3) with the *Bmal1-luc* report gene, monitoring in real-time *Bmal1* expression period and amplitude changes in these two types of cells under different T-cycle DEX stimuli. The results were consistent with the behavioral observations, indicating that BALB/c mice exhibited a wider range of adaptability to external periodic stimuli. Subsequently, we discovered that BALB/c mice adapted faster to the +6 shift while showing lower amplitudes and activity levels in DD and LD, consistent with previous studies suggesting poorer coupling rhythms in BALB/c mice [11,20]. At the genetic level, we verified through RNA and protein experiments that the expression of *Bmal1* and *Npas2* in BALB/c mice was significantly lower than in C57BL/6 mice, both at the cellular and tissue levels. Especially, the DEGs in both the intestine and lung included *Npas2* by acquiring RNA-seq data from GEO, combined with the behavioral rhythm of *Npas2* knockout mice [18,21], revealed that BALB/c mice can rapidly entrain to LD shift, which is most likely attributed to a rarely noticed circadian clock gene *Npas2*, providing guidance for future research in selecting circadian clock mouse models.

Given that each cell possesses a circadian rhythm system, most in vitro studies have concentrated on the impact of a single treatment on the phase shift of the biological clock [22], while few studies have utilized repetitive stimuli, for instance, 24-h stimulation cycles spanning several days. This is because repeated stimuli may induce medium alterations that result in the resetting of the circadian phase [23]. It has been reported that three cycles of repetitive stimulation using a luciferase reporter can evaluate the entrainment capacity of cellular clocks [24]. Although we tested the aftereffects of DEX-stimulated cells, these effects are sufficient to represent the changes in cell rhythms during the T cycles. Previous research has shown that three cycles of DEX stimulation are adequate to evaluate the entrainment ability [24]. Moreover, Yool Lee et al. demonstrated that the phase shifts in cells after DEX treatment persist for at least one week, confirming that cells exhibit sustained adaptability to periodic stimulation over time [25]. Therefore, it is completely feasible to monitor the bioluminescence rhythm of *Bmal1* after treating 7 cycles to the cells and analyzing the entrainment of cells to external periodic stimuli. Our results show that after stimulation with different T cycles, BALB/3T3 cells (T22–28) have a wider range of adaptability than MEF (T24–26) cells.

In a study of behavioral rhythms in mice entraining to LD shifts adaptability, most of them focused on behavior actograms and phase change statistics [9,20]. However, we combined two methods to provide a more precise analysis of the rate of entraining to phase shift in mice. Our study reveals that consistent with the cellular findings, BALB/c mice exhibit a more flexible adaptation to LD shifts compared to C57BL/6 mice, which aligns with previous research [11,26]. This may be attributed to the weaker coupling ability of the circadian rhythm in BALB/c mice, resulting in greater adaptability to different light–dark cycles in terms of behavioral rhythm [27].

We detected the expression of classic clock genes in the cells and tissues of C57BL/6 and BALB/c mice, then collected and analyzed the differentially expressed genes in existing tissues of WT C57BL/6 and BALB/c mice from the GEO database. The results indicate significant differences in *Npas2* expression between C57BL/6 and BALB/c mice. The central tissue of the brain, which controls behavioral rhythm, was also analyzed. Of course, we cannot rely solely on identifying DEGs to determine the genes that cause behavioral rhythm differences between BALB/c and C57BL/6 mice, and further mechanism analysis should be explored in subsequent research. There have been some reports on the molecular mechanisms that affect the ability of mice to entrain jet lag; previous studies have indicated that vasoactive intestinal peptide (VIP) is an essential neuropeptide. Exposure of SCN to VIP reduces intercellular synchronization, thereby accelerating the adaptation of mice to jet lag [28,29]. The lack of vasopressin receptors V1a and V1b also leads to the mice adapting to jet lag faster [16]. However, the DEGs from GEO data in C57BL/6 and BALB/c mice did not detect any changes in these two genes. Nevertheless, we discovered that the expression of *Npas2* in BALB/c mice was significantly lower than that in C57BL/6 mice. In the meantime, studies have found that C57BL/6 mice lacking *Npas2* have shorter FRP and faster adaptation to jet lag [18,21], which are more aligned with the characteristics of the circadian behavior rhythm of BALB/c mice.

NPAS2 forms a heterodimer with the transcription factor BMAL1. The transcriptional targets of NPAS2-BMAL1 include periods (PER1, PER2, and PER3) and cryptochromes (CRY1 and CRY2), which regulate the biological rhythms of mammalian organs [30,31]. NPAS2-BMAL1 also activates the transcription of circadian genes by binding to the E-box (CACGTG) sequence, participating in the circadian feedback loop. Circadian genes are believed to be primarily expressed in the SCN, but new evidence suggests that *Npas2* may also play a significant role in peripheral tissues [14]. Our study proved that compared with C57BL/6 mice, BALB/c mice match to LD shift more rapidly, and the expression level of *Npas2* is significantly reduced, combined with GEO database analysis and previous studies [21], suggesting that *Npas2* may be a key gene regulating their behavioral rhythm adaptability. This provides constructive insights for subsequent research on selecting model mice for circadian rhythm studies. However, there are no significant differences in the SCN structure between these two strains [32]. The differences primarily lie in genetics, as evidenced by genome-wide complex trait and epistasis analyses revealing numerous loci differences on different chromosomes and many undiscovered additional clock genes [33,34,35]. This indicates that the factors regulating the adaptation of behavioral rhythm in BALB/c mice to external light cycles are multifaceted and not entirely controlled by a single gene.

Several limitations to our study should be acknowledged. First, the T cycles of periodic stimulation were not dense enough to capture more nuanced entrainment patterns. Additionally, our investigation into the regulatory mechanisms of *Npas2* was not comprehensive, as we did not explore the effects of *Npas2* overexpression in BALB/c mice and their cells or knocking down this gene in C57BL/6 mice and cells. Addressing these gaps can be a key direction for future research. Moreover, our study primarily focused on gene expression levels without accounting for phase differences between tissues. Despite the inertia effects observed in after-effects following DEX stimulation [24], other factors, such as environmental conditions or stress, may still influence after-effects, limiting their ability to fully reflect preceding entrainment. Lastly, using primary cell lines constructed with a clock reporter would likely yield more accurate insights than immortalized cell lines.

Although there are limitations to our study, our findings provide important insights into the genetic and cellular mechanisms underlying circadian adaptability, laying the groundwork for future studies that will further elucidate the role of *Npas2* and other clock genes in circadian regulation.

## 4. Materials and Methods

### 4.1. Animals

Male mice (6–8 weeks of age), including C57BL/6 and BALB/c mice, were kept under a normal 12 h light–12 h dark cycle (LD, lights on 8:00 (Zeitgeber time 0, ZT0); lights off 20:00 (ZT12)) for at least 2 weeks before the experiment. Light intensity was kept at about 100 lux in the light phase. Mice could freely access normal food and water at room temperature at 23 ± 2 °C. All animal research procedures were approved by the Institutional Animal Care and Use Committee of Dalian University of Technology, Shanghai University of Medicine & Health Sciences.

### 4.2. Cell Culture and Stable Transfection with the Bmal1-Luc Reporter

MEF (Shanghai Hongshun Biotechnology Co., Ltd., Shanghai, China) and BALB/3T3 (Chinese Collection of Authenticated Cell Cultures, Wuhan, China) cells were cultured in Dulbecco’s modified Eagle’s medium with 4500 mg/L glucose (DMEM, Gibco, Thermo Fisher Scientific, Waltham, MA, USA). The medium was supplemented with 10% fetal bovine serum (FBS, Sigma, St. Louis, MO, USA), and 100 U/mL penicillin-streptomycin (Sigma, St. Louis, MO, USA) in a humidified 5% CO_2_ incubator at 37 °C.

For transfection, the MEF and BALB/3T3 cells were stably transfected with the *Bmal1-luc* reporter gene for real-time recorded expression rhythm of *Bmal1* according to the previous method [24,36]. Specifically, 20 μg/mL of the three-plasmid system viral vector system pSPAX2 pHCMV-VSV-G (Heyuan Biotechnology Co., Ltd., Shanghai, China), including the *Bmal1-luc* region (Addgene, #46824, Watertown, MA, USA) was transfected into 10^5^ cells. After 72 h, the cells were cultured in a selective medium containing 1 μg/mL of puromycin (Sigma, St. Louis, MO, USA) for 2 weeks, and the cells stably expressing the *Bmal1-luc* were obtained.

### 4.3. Dexamethasone Periodic Treatment and Bioluminescence Analysis

The MEF and BALB/3T3 cells expressing luciferase were given with DEX (100 nm, Sigma, USA) for the indicated time to evaluate the entrainable range of the two types of cells to external treatment cycles based on the previously described method [24,25]. Specifically, the period of the stimulation cycles is denoted as T. T cycles < 24 h (i.e., T = 16, 18, 20, 22) and ≥ 24 h (i.e., T = 24, 26, 28, 30) for 7 sustained cycles. After the last DEX treatment (for free-running was a single DEX treatment), 0.1 mM D-luciferin potassium salt (Promega, Fitchburg, Madison, WI, USA) was added to the culture medium stated above, and the cells were placed in a LumiCycle (Actimetrics, Wilmette, IL, USA) to record bioluminescence activity that was monitored at 10-min intervals. As previously described [24,37], the 24-h running average was subtracted from the raw data to detrend the bioluminescence data, and the period and amplitude of cells were determined by the LumiCycle software (version: 3.002) program (Actimetrics, Wilmette, IL, USA).

### 4.4. Wheel Running Activity Recording and Analysis

C57BL/6 and BALB/c mice were individually housed in cages equipped with running wheels, which were placed in ventilated and light-proof boxes (Probecare, Wuhan, China). The locomotor activities were continuously recorded at 1-min intervals and analyzed using ClockLab software (version: 6.1.02) (Actimetrics, Wilmette, IL, USA), which automatically created the double-plotted actograms by recording the wheel rotations. To thoroughly analyze the 1-day (24-h) activity patterns of mice in LD and DD conditions, the mean activity profiles were generated by ClockLab, and running wheel counts during daytime, nighttime, and across the entire day were calculated separately. FRP under DD was determined through chi-square periodogram analysis [38]. The circadian amplitude reflects the robustness of circadian behavior and can be obtained through fast Fourier transform (FFT)-relative power [39], which identified the highest value of the relative spectral power for the circadian period to assess the strength of rhythmicity. In addition, the onset time of daily activity is quantified using an internal algorithm in ClockLab. To measure the re-entrainment speed of mice to the 6-h advance phase shift, we employed two methods: (1) the days to reach 50% phase shift (PS50) [40]; (2) the difference between the onset of activity and the lights off (phase angle of entrainment) < 0.5 h for at least three consecutive days [41]. The approach of quantifying the magnitude of phase shifts by releasing mice into DD following an LD shift aligns with previous research [16].

### 4.5. Data Visualization and Identification of DEGs

We utilized the GEO2R tool to analyze DEGs in five datasets (GSE7814, GSE34010, GSE111155) respectively derived from whole brains, intestines, and lungs of WT C57BL/6 and BALB/c mice in the GEO database (https://www.ncbi.nlm.nih.gov/geo/ Accessed on 25 February 2024). The volcano plots were generated for each dataset from the hiplot database (https://hiplot.com.cn/home/index.html. Accessed on 7 March 2024) [42]. Metascape was employed for clustering and GO enrichment analysis of DEGs [43]. The results of the bubble plot were visualized and displayed using the hiplot database. Statistically significant DEGs were defined as those with a logFC (fold change) of ≥ |1.2| and a Bonferroni-corrected *p*-value of < 0.05.

### 4.6. RNA Extraction and qRT-PCR

MEF and BALB/3T3 cells were treated with 100 nM dexamethasone (Sigma, USA) for synchronization, and were collected every 4 h during the following 48 h after 24-h synchronization of the last treatment. C57BL/6 and BALB/c mice were sacrificed at ZT0 (the peak of *Npas2* expression) and liver, hypothalamus, intestine, lung, brown adipose tissue (BAT), heart, and skin were collected. Total RNAs were extracted with TRIzol reagent (Thermo Fisher Scientific, USA), adhering to the manufacturer’s protocol, and 1 μg of total RNA was reversed to cDNA using the reverse transcription kit (Tiangen Biotechnology Co., Ltd., Beijing, China) on LightCycler 96 (Roche, Basel, Switzerland). Sequences of PCR primers are shown in Table S2.

### 4.7. Western Blot

Total tissue proteins were lysed utilizing RIPA lysis buffer (Beyotime Biotechnology Co., Ltd., Shanghai, China), and protein concentration was determined with Pierce™ BCA Protein Assay Kit (Thermo Fisher Scientific, USA). According to the previous description [44], 10% SDS-PAGE was used for immunoblotting of proteins, and then the proteins were transferred to the nitrocellulose membrane. After being blocked with 5% skimmed milk at room temperature for 1 h, the membrane was incubated overnight at 4 °C with anti-BMAL1 (Abcam, ab93806, Cambridge, UK), anti-NPAS2 (ABclonal, A16930, Wuhan, China), and anti-β-actin (Proteintech, 20536-1-AP, Rosemont, IL, USA) primary antibodies and incubated with relevant horseradish peroxidase-conjugated secondary antibodies at room temperature for 1 h. The immunoblots were detected using the Chemiluminescence Imaging System (Tanon, Shanghai Tianneng Technology Co., Ltd., Shanghai, China) and analyzed with ImageJ software (version: 1.8.0).

### 4.8. Statistical Analyses

The data were analyzed by an independent sample *t*-test and a two-way analysis of variance (ANOVA) performed with GraphPad Prism 9.0 software (GraphPad Software, San Diego, CA, USA). All results were expressed as mean ± standard error mean (SEM). * *p* < 0.05, ** *p* < 0.01, *** *p* < 0.001.

## Data Availability

The data presented in this study are openly available in GEO datasets.

## Supplementary Materials

The following supporting information can be downloaded at: https://www.mdpi.com/article/10.3390/ijms251910404/s1.
