# Supplementary material for: Adaptive Differences in Cellular and Behavioral Responses to Circadian Disruption between C57BL/6 and BALB/c Strains
Source: Int J Mol Sci. 2024 Sep 27;25(19):10404. doi: 10.3390/ijms251910404 (PMC11476807; doi:10.3390/ijms251910404)
Supplement: Supplementary file 1 [file ijms-25-10404-s001.zip › supplement/ijms-3134466-supplementary.docx]

**Supplement Figures**

**Figure S1**. The mRNA expression of core clock genes in the liver, BAT, and heart of C57BL/6 and BALB/c mice. (A) *Bmal1*; (B) *Per1*; (C) *Per2*; (D) *Clock*; (E) *Cry1*; (F) *Cry2*; (G) *Nr1d1*; (H) *Npas2* (n = 4; * p < 0.05, ** p < 0.01, *** p < 0.001).

**Figure S2.** GEO data analysis in whole brain, intestine, and lung of C57BL/6 and BALB/c mice. (A and B) Volcano plot (A) and GO analyses (B) of DEGs in BALB/c mice compared to C57BL/6 mice in the whole brain (n = 8). (C and D) Volcano plot (C) and GO analyses (D) of DEGs in BALB/c mice compared to C57BL/6 mice in the intestine (n = 4). (E and F) Volcano plot (E) and GO analyses (F) of DEGs in BALB/c mice (n = 5) compared to C57BL/6 mice (n = 6) in the lung.
