# Supplementary material for: Adaptive Differences in Cellular and Behavioral Responses to Circadian Disruption between C57BL/6 and BALB/c Strains
Source: Int J Mol Sci. 2024 Sep 27;25(19):10404. doi: 10.3390/ijms251910404 (PMC11476807; doi:10.3390/ijms251910404)
Supplement: Supplementary file 1 [file ijms-25-10404-s001.zip › supplement/Table.pdf]

Table S1. The bioluminescence period of MEF and BALB/3T3 cells expressing the Bmal1-Luc reporter gene under T cycles by dexamethasone (DEX) stimulation.

	**Free**	T16	T18	T20	T22	T24	T26	T28	T30
MEF	**23.00 ± 0.10**	$22.50 \pm 0.06^{***}$	$22.90 \pm 0.06^{***}$	$22.80 \pm 0.10^{**}$	$22.83 \pm 0.03^{*}$	23.83 ± 0.13	26.00 ± 0.12	$23.73 \pm 0.19^{***}$	$25.10 \pm 0.10^{***}$
BALB/3T3	**22.43 ± 0.03**	$22.07 \pm 0.09^{\#\#\#}$	$21.00 \pm 0.12^{\#\#\#}$	$20.73 \pm 0.38^{\#\#}$	22.37 ± 0.03	23.67 ± 0.12	26.10 ± 0.12	28.03 ± 0.13	$25.87 \pm 0.19^{\#\#\#}$
P-value	< 0.01	0.01	< 0.001	< 0.01	< 0.001	0.41	0.57	< 0.001	0.02

Table S1. The bioluminescence period of MEF and BALB/3T3 cells expressing the Bmal1-Luc reporter gene under T cycles by dexamethasone (DEX) stimulation. The P-value represents the significance of the bioluminescence periods of MEF vs BALB/3T3 cells in different T-cycles. $^{***}p < 0.001$, $^{**}p < 0.01$, vs T cycle in MEF and $^{\#\#\#}p < 0.001$, $^{\#\#}p < 0.01$ vs T cycle in BALB/3T3.

Table S2. List of primers used for qRT-PCR.

**Name**	**Sequence**
Mouse-Ogt (Forward Primer)	5'-GACGCAACCAAACTTTGCAGT-3'
Mouse-Ogt (Reverse Primer)	5'-TCAAGGGTGACAGCCTTTTCA-3'
Mouse-Dbp (Forward Primer)	5'-CTGGCCCGAGTCTTTTTGC-3'
Mouse-Dbp (Reverse Primer)	5'-CCAGGTCCACGTATTCCACG-3'
Mouse-Pten (Forward Primer)	5'-GACGCAACCAAACTTTGCAGT-3'
Mouse-Pten (Reverse Primer)	5'-GCGGTGTCATAATGTCTCTCAG-3'
Mouse-Bmal1 (Forward Primer)	5'-AAGAGGCGTCGGGACAAAAT-3'
Mouse-Bmal1 (Reverse Primer)	5'-TCTGTGTATGGGTTGGTGGC-3'
Mouse-Per1 (Forward Primer)	5'-AACGGGATGTGTTTCGGGGTG-3'
Mouse-Per1 (Reverse Primer)	5'-AGGACCTCCTCTGATTCGGCA-3'
Mouse-Per2 (Forward Primer)	5'-TGATCGAGACGCCTGTGCTCG-3'
Mouse-Per2 (Reverse Primer)	5'-CTCCACGGGTTGATGAAGCTG-3'
Mouse-Clock (Forward Primer)	5'-CACTCTCACAGCCCCACTGTA-3'
Mouse-Clock (Reverse Primer)	5'-CCCCACAAGCTACAGGAGCAG-3'
Mouse-Cry1 (Forward Primer)	5'-AGCGCAGGTGTCGGTTATGAG-3'
Mouse-Cry1 (Reverse Primer)	5'-ATAGACGCAGCGGATGGTGTC-3'
Mouse-Cry2 (Forward Primer)	5'-TGTCCCTTCCTGTGTGGAAGA-3'
Mouse-Cry2 (Reverse Primer)	5'-GCTCCCAGCTTGGCTTGA-3'
Mouse-Nr1d1 (Forward Primer)	5'-CCCACATACTTCCCACCATC-3'
Mouse-Nr1d1 (Reverse Primer)	5'-CACAGTAGCACCATGCCATT-3'
Mouse-Npas2 (Forward Primer)	5'-CGTCGGGACCAGTTCAATGTT-3'
Mouse-Npas2 (Reverse Primer)	5'-AGCACGGTGGTTTTGTCCAT-3'
Mouse-18s (Forward Primer)	5'- ACCGCAGCTAGGAATAATGGA-3'
Mouse-18s (Reverse Primer)	5'- GCCTCAGTTCCGAAAACCA-3'
